# Parallelism in the brain's visual form system

**DOI:** 10.1111/ejn.12371

**Published:** 2013-10-07

**Authors:** Yoshihito Shigihara, Semir Zeki

**Affiliations:** Wellcome Laboratory of Neurobiology, University College LondonGower Street, London, WC1E 6BT, UK

**Keywords:** form perception, hierarchical model, orientation selective cells, prestriate cortex, striate cortex

## Abstract

We used magnetoencephalography (MEG) to determine whether increasingly complex forms constituted from the same elements (lines) activate visual cortex with the same or different latencies. Twenty right-handed healthy adult volunteers viewed two different forms, lines and rhomboids, representing two levels of complexity. Our results showed that the earliest responses produced by lines and rhomboids in both striate and prestriate cortex had similar peak latencies (40 ms) although lines produced stronger responses than rhomboids. Dynamic causal modeling (DCM) showed that a parallel multiple input model to striate and prestriate cortex accounts best for the MEG response data. These results lead us to conclude that the perceptual hierarchy between lines and rhomboids is not mirrored by a temporal hierarchy in latency of activation and thus that a strategy of parallel processing appears to be used to construct forms, without implying that a hierarchical strategy may not be used in separate visual areas, in parallel.

## Introduction

Ever since the discovery of orientation-selective (OS) cells in V1 (the primary visual cortex; Hubel & Wiesel, 1962, 1977) it has been supposed that they constitute the physiological building blocks for the elaboration of perceived forms and that, consequently, the brain analyses the visual world in hierarchical steps, each step constituting a more complex level of analysis than the preceding one (Hubel & Wiesel, 1962). This supposition derives from the observation that OS cells have increasingly complex properties (Hubel & Wiesel, 1962), with simpler cells feeding into more complex ones in the same (Hubel & Wiesel, 1962) or in a ‘higher’ visual area (Hubel & Wiesel, 1965). In the macaque brain, OS cells are prominent constituents in reciprocally connected visual areas V1, V2 and V3 (Zeki, 1978a,1978b; Zeki & Shipp, 1988; Economides *et al*., 2011). Although the anatomical pathways linking these areas with one another and with subcortical stations are, inevitably, less clear in the human brain, the assumption is that there, too, the properties of ‘higher’ form areas are ultimately traceable to the OS cells of V1 (e.g. Riesenhuber & Poggio, 1999), and oriented lines have indeed been determined to be good stimuli for activating these human equivalents (Tootell *et al*., 1988; Kourtzi *et al*., 2003; Kamitani & Tong, 2006; Yacoub *et al*., 2008; Tong *et al*., 2012).

As a global strategy for analysing the visual world, the hierarchical model was significantly modified by the discovery of parallel processing and functional specialisation for processing different visual attributes in different areas of the primate visual brain (Zeki, 1976, 1978a,1978b, 1991; Van Essen *et al*., 1990). But, given the parallel outputs in monkey from the pulvinar and the lateral geniculate nucleus to both V1 and areas of the prestriate cortex (Cragg, 1969; Benevento & Rezak, 1976; Fries, 1981; Yukie & Iwai, 1981; Baldwin *et al*., 2012), we wanted to learn whether a parallel strategy is also used within the visual form system, widely considered to be hierarchically organised (e.g. Van Essen *et al*., 1990). As a first step in this enquiry, we thought it interesting to learn whether forms of increasing complexity constituted from the same elements (lines) will activate the striate and prestriate visual cortex with the same or different latencies. To do so, we used magnetoencephalography (MEG) to measure visual evoked responses when humans viewed two forms (lines and rhomboids) of increasing complexity. If the form perception system is organised hierarchically, more complex forms such as rhomboids should activate visual cortex with longer latencies than the simpler ones (lines) from which they are built.

## Materials and methods

### Subjects and study design

Twenty right-handed healthy adult volunteers (10 female, mean age 28.2 years) took part; none had a history of neurological or psychiatric disorders. Written informed consent was obtained from all and the study, which conforms to Code of Ethics of the World Medical Association (Declaration of Helsinki; printed in the British Medical Journal 18 July 1964), was approved by the Ethics Committee of University College London.

### Stimuli and task

**Figure 1:**
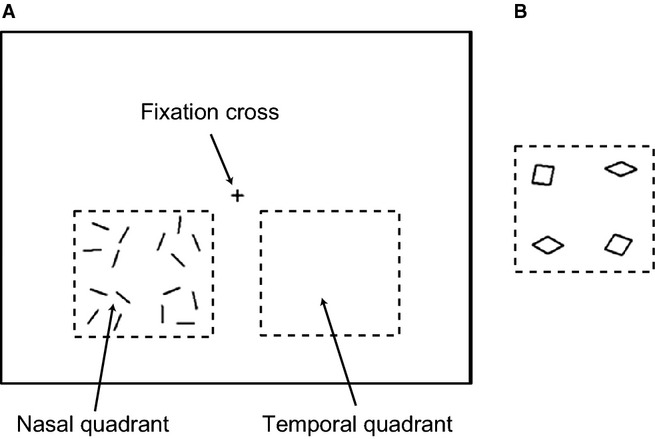
Stimuli consisting of (A) 16 lines or (B) four rhomboids, made of the same components, appeared in lower left (nasal) or right (temporal) quadrants. Subjects viewed the central fixation cross monocularly with the right eye; they were instructed to report changes in the fixation cross by pressing a button with their right index finger. Squares drawn by dashed lines represent areas in which the stimuli were projected.

Stimuli and trigger signals were generated using cogent 2000 and cogent graphics (http://www.vislab.ucl.ac.uk/cogent.php) toolboxes running in matlab (MathWorks, Natick, MA, USA). Stimuli were rear-projected onto the screen by a projector (RM-MSX21G; Victor Company of Japan, Kanagawa, Japan), with a resolution of 1024 × 768 pixels at 60 Hz, and trigger signals were recorded for the MEG system through an IEEE 1284 connection. The delay between the trigger signal and stimuli projection (17 ms) was corrected during data processing. Subjects viewed the stimuli monocularly with the right eye (a patch covered the left eye) at a distance of between 40 and 60 cm, according to the individual's comfort and clarity of vision. Because previous studies have reported some asymmetries in the visual system between nasal and temporal visual hemi-fields (Fahle & Schmid, 1988; Sylvester *et al*., 2007; Silva *et al*., 2010), and to avoid cancellation effects which can occur with MEG when current sources are present on opposing banks of the calcarine sulcus (Portin *et al*., 1999), stimuli were displayed separately in either the lower left (nasal) or lower right (temporal) quadrants of the visual field, between 1.3° and 12.3° below the fixation cross and 2.3° to 13.8° on either side. Stimuli consisted of 16 separate white lines or four rhomboids made of the same 16 lines (Fig. 1). The lines subtended 1.5 × 0.1°, and a white fixation cross subtending 1.0 × 1.0° was projected in the centre of the screen. The vertices of the rhomboids varied from 18 to 162°. To reduce participants’ eye movements and maintain their attention levels the fixation cross periodically increased its vertical size from 1.0 to 1.2° for durations of 200 ms, which the subjects were instructed to report by pressing a button with their right index finger.

### Scanning details

In all 20 subjects, MEG data was recorded continuously using a 275-channel CTF Omega whole-head gradiometer (VSM MedTech, Coquitlam, Canada). Data were sampled at 1200 Hz with a 200-Hz low-pass filter. Subjects were fitted with localiser coils at the nasion and 1 cm anterior to the left and right tragus to monitor head position during the recording sessions. Gaze position and blinking were monitored by an EyeLink 1000 eye tracker (SR Research Ltd., Ontario, Canada) except in five subjects where there were technical problems. Eye blinking data was also used for artifact detection during the averaging process.

### Signal-to-noise ratio

We departed from the use of stimuli such as checkerboards, grating patterns and flashing lights, which are known to be efficient stimuli for producing visual evoked responses (Regan, 1988), and used instead 16 white lines, presented either singly or arranged into rhomboids, to address our question regarding the latency of activation produced by forms of increasing complexity. We were especially interested in the very early (i.e. initial) components of event-related magnetic fields (ERFs) which have a very low signal-to-noise ratio (SNR). We therefore used three to four times as many epochs for each condition as is customary. Indeed, SNR at the initial response was such that it was not practical to draw conclusions from single subjects, which is why we present results from the group analysis. The experiment consisted of six 5-min runs. Each run comprised 300 stimulus presentations (roughly equal proportions of lines and rhomboids presented in nasal and temporal quadrants) in a pseudo-randomised sequence, each lasting 200 ms with a randomly varying interstimulus interval of 600–800 ms, because when the interstimulus interval is varied, the SNR is improved through reduction in the background wave.

### Data processing

**Figure 2:**
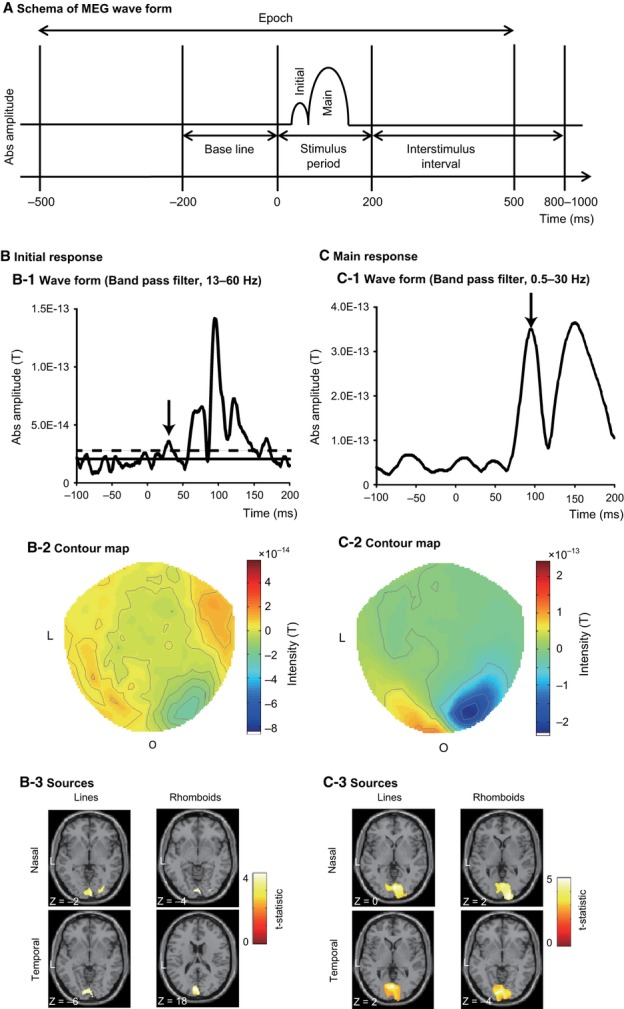
Two sequential ERFs. (A) Schema of MEG waveform. MEG data was divided into 1000-ms epochs, each commencing 500 ms before a stimulus onset. The 200-ms period preceding stimulus onset formed a baseline signal condition. An initial response was identified at ∼ 40 ms after stimulus onset, followed by a main response at ∼100 ms. (B) The initial response at 40 ms. (B-1) Absolute amplitude time-course of ERF measured by occipital sensors for the initial response (bandpass filter, 13–60 Hz) for a typical subject. The arrow indicates a peak of the initial response. Solid and dashed lines show the baseline average and average + 2 SD respectively. (B-2) Contour map at the initial response latency (averaged between 27 and 44 ms after stimulus onset) showing a source in right occipital cortex. (B-3) Statistical parametric map of estimated source locations of ERFs for group-level analysis (between subjects) superimposed on a standard brain image, broken down by stimulus form and display quadrant for the initial response. (C) Main response at 100 ms. (C-1) Absolute amplitude time-course for the main response (bandpass filter, 0.5–30 Hz) for a typical subject. The arrow indicates a peak of the main response. (C-2) Contour map at the main response latency (95 ms after stimulus onset) showing a source in right occipital cortex. (C-3) Statistical parametric map of estimated source locations of ERFs for group-level analysis superimposed on a standard brain image, broken down by stimulus form and display quadrant for main response. L, left; O, occipital. This figure displays the waveforms and contour maps for a typical subject when viewing line stimuli presented in the nasal quadrant. A display threshold of *P*_unc._ < 0.001 is used in B3 and C3.

**Figure 3:**
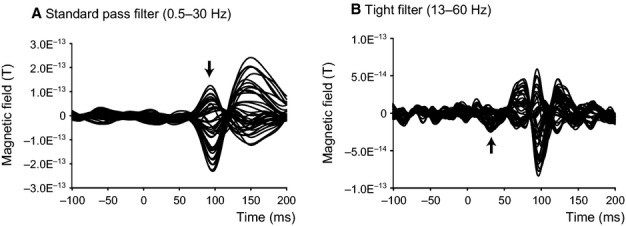
Typical MEG wave forms processed by different filters. (A) Record when a standard filter (bandpass, 0.5–30 Hz) was applied; a main response (P100 m) can be identified at ∼100 ms but the initial response is not clear. (B) Record when a tight filter (bandpass, 13–60 Hz) was applied; now the initial response (at ∼40 ms) can be identified, but now the main response (P100 m) has three peaks, two of which were filter artifacts of the main response. By using both filters, one can distinguish the two. Arrows indicate (A) main and (B) initial responses.

Data were analysed offline using spm-8 (Wellcome Trust Centre for Neuroimaging, London, UK; http://www.fil.ion.ucl.ac.uk/spm) and divided into 1000-ms epochs, each starting 500 ms before a stimulus onset (Fig. 2A). Our analysis software (SPM) requires a 500-ms pre-stimulus period, but as this could include the ERF from the previous stimulus we selected the −200-to +200-ms region for actual analysis. Epochs affected by blink artifacts (detected using the eye-tracker and also by manual inspection of the raw signal data) were discarded and the remaining ones averaged in each condition, baseline-corrected and filtered. The signal during the 200-ms period preceding stimulus onset was used as a baseline. ERFs showed two different sequential responses: a main response (P100 m; Tobimatsu & Celesia, 2006) ∼100 ms and a very early response (initial response; Inui *et al*., 2006) ∼40 ms after stimulus onset. Two different filter settings were applied to isolate the responses: a standard 0.5–30 Hz bandpass filter for the main response and a tight 13–60 Hz bandpass filter for the initial one (Fig. 3). Although the standard setting is suitable for identifying the peak of the wave form at ∼100 ms it is not reliable for identifying the initial response, which may be obscured by low-frequency components. Previous studies have shown that the P100 and N145 responses are related to alpha and theta waves respectively (Klimesch *et al*., 2004). Thus, in theory, the initial response should consist of higher frequency components, and suppression of low-frequency (alpha and theta) components should highlight this initial component. As mentioned above, a standard filter (0.5–30 Hz) was used for analysing the main response (P100 m) due to its relation to alpha waves. Sensor level analysis was used to measure amplitudes and identify the latencies of ERFs, and source-level analysis was used for source estimation; the two analyses were done separately for the initial and main responses. We restricted our dynamic causal modeling (DCM) analyses to the time window around the initial response, where differences between sequential and parallel processing would be expected to be most evident.

### Outline of analysis

We had three specific goals in this study: to determine amplitude differences at sensor level, to determine differences in source distribution, and to determine latency differences between line and rhomboid stimulation, especially for the initial response. The interpretation of the results relies critically on the initial response, which occurs ∼40 ms after stimulus onset and which can be rather weak and difficult to detect. Our ability to detect the initial response was confirmed in three ways: first, we were able to identify the P100 m response in every subject and for every tested condition, thus confirming the correct operation of the recording equipment; second, we used a supplementary (laterality) check to determine that we were getting stronger responses from the hemisphere contralateral to the stimulated hemi-field and confirmed that this was indeed so at the group level and at ∼40 ms; third, we confirmed that, at 26 ms after stimulus onset, the absolute amplitude of visual response was greater than the baseline average + 2 SD. We restricted ourselves to the group level because the SNR in individual subjects was too noisy. With these checks we analysed the MEG data for amplitude and latency at the sensor level, followed by source localisation and a DCM analysis at the source level. We did not analyse source waveforms because their peaks were not clear enough, due to their low SNR (see above).

We concentrated our analysis on the initial response because the main response (P100 m) is complicated due to feedback. Murray *et al*. (2002) and Fang *et al*. (2008) showed that feedback can inhibit activity in V1, while Lamme & Roelfsema (2000) showed that cells in V1 can process low-level features at 40 ms after stimulation but also process more global features at 100 ms after onset. We therefore used an event-related analysis which isolates the initial response (Inui *et al*., 2006).

### Amplitude analysis

We compared ERF amplitudes between lines and rhomboids, using eighteen left occipital (MLO 11-53) and nineteen right occipital (MRO 11-53) sensors. Absolute amplitude was calculated as the difference between maximum and minimum magnetic fields within sensor sets at each time bin. The right occipital sensors were used to calculate amplitudes at intervals of 0.833 ms (67 time bins) between 15 and 70 ms after stimulus onset over all twenty subjects, using a tight filter. As we were making multiple comparisons we only accepted a difference when the *t*-tests for four or more consecutive time bins gave the same result (Liu *et al*., 2002). Because the individual *t*-test threshold was *P* < 0.05, this was a more conservative measure than applying a Bonferroni correction. Guthrie & Buchwald (1991) describe a threshold criterion to correct for sampling autocorrelation in waveforms but we could not employ that method because our initial response was too short and of low amplitude due to its high frequency. Instead we confirmed the initial response, separately for nasal and temporal stimulation, in each subject, by graphical inspection of the signal waveform.

### Latency analysis

To analyse response latencies we used all forty sensors in the occipital area (channels MLO 11-53, MRO 11-53 and MZO 01-03) to improve the SNR for the responses. Latency was defined as the instant of peak field amplitude within a specified time window after stimulus onset (15–70 ms for the initial response and 70–160 ms for the main response). The main response was clear but the initial response not so because the amplitude was not large, making it difficult to distinguish from the noise of the background alpha wave. We therefore employed the tighter bandpass filter mentioned above to reduce the alpha wave effect and specified the additional criterion (for latency analysis only) that the peak amplitude should be greater than the mean + 2 SD of the baseline for four or more consecutive time bins (Fig. 2, b1; at least 3 ms; Noguchi & Kakigi, 2006). Although only 41 out of 80 measurements satisfied these stringent latency criteria (see Table 1), we found corroboratory evidence in the ERF contour maps which displayed clear characteristic dipole patterns in the occipital area in most cases (74 out of 80).

**TABLE 1:** Latencies of event-related magnetic fields

		Nasal		Temporal	
Responses	Stimuli	*N*	Mean ± SD	*N*	Mean ± SD
Initial	Line	14	31.8 ± 7.5	9	41.9 ± 10.2
	Rhomboid	8	37.5 ± 5.9	10	36.2 ± 8.1
Main	Line	20	100.2 ± 13.9	20	96.7 ± 14.0
	Rhomboid	20	92.3 ± 11.5	20	99.7 ± 17.6

Values for peak latencies broken down by initial and main responses, stimulus form and display quadrant. Data for 20 subjects. Not all subjects gave an initial response. *N*, the number of subjects who showed a response which met the criteria described in Materials and Methods.

We used a two-way (quadrant × form) anova for repeated measurements to compare the main response latencies for nasal and temporal quadrant presentations of lines and rhomboids. To perform a similar comparison for the initial response latencies we used a univariate two-way (quadrant × form) anova because only 50% of the data met criteria for the initial response (see above for the further explanation).

### Source analysis

SPM-8 has three options for source localisation as a distributed source model: minimum norm (Hämäläinen & Ilmoniemi, 1984), LORETA (Pascual-Marqui *et al*., 1994) and Multiple Sparse Priors (MSP; Greedy Search; Mattout *et al*., 2005; Friston *et al*., 2008). We used the MSP for source estimation at single-subject level (1st level) for both the initial and main responses, using individual subject anatomical scans because MSP has relatively high spatial resolution in those three options. For the main response, the sources for each of the four conditions (lines and rhomboids presented in nasal and temporal quadrants) were estimated within a time window of ±5 ms of the peak latencies obtained for each condition in each individual subject. Because the initial response was not clear in all subjects, the time window for source localisation was fixed at 27–44 ms, which was the mean ± SD of the initial response in those subjects and for all conditions where it was clearly expressed. Model inversion was performed within each time window. High-and low-pass filters for source inversions were set at 0 and 48 Hz respectively, although a standard and a tight filter had already been applied to the data prior to the source inversion. We did not select any prior sources, nor was the solution constrained to any region within the whole brain volume.

Source images for each condition at each response were smoothed using a Gaussian smoothing kernel of 8 × 8 × 8 mm and taken to the group level (between subjects; Koelewijn *et al*., 2011). Gaussian random field theory controlled for multiple comparisons in 3-D space (source space; Kiebel & Friston, 2002; Kilner *et al*., 2005; Friston *et al*., 2008). Two types of statistical map were constructed: for each condition (vs. baseline) using a one-sample *t-*test and between two conditions (i.e. lines vs. rhomboids) by paired *t-*test.

In the Results section we report the source locations of peak level activations at a significance threshold of *P*_unc._ < 0.001. Although we are reporting uncorrected statistics, the existence of these ERFs was independently established at a statistically significant level by the laterality check, amplitude analysis and latency analysis (see above), so the lower statistical threshold only applies to the locations of the peaks, not the existence of the responses. The visual area for each peak was identified using the SPM Anatomy toolbox (http://www2.fz-juelich.de/inm/index.php?index=194), with striate and prestriate cortices corresponding to Brodmann Areas 17 and 18 respectively. If multiple peaks were present within an area, the maximum intensity peak was chosen for analysis.

### DCM

We applied DCM (Friston *et al*., 2003; Garrido *et al*., 2007; Kiebel *et al*., 2007) by applying SPM-8 to our MEG results, to compare different models of how brain areas may interact in our experiments. For this we focused on the initial response alone, as it is during this very early processing phase that differences between the various models are likely to be expressed. We repeated the DCM analysis for the four separate conditions (lines and rhomboids, nasal and temporal quadrants) and then combined the results of the nasal and temporal quadrant presentations.

**Figure 4:**
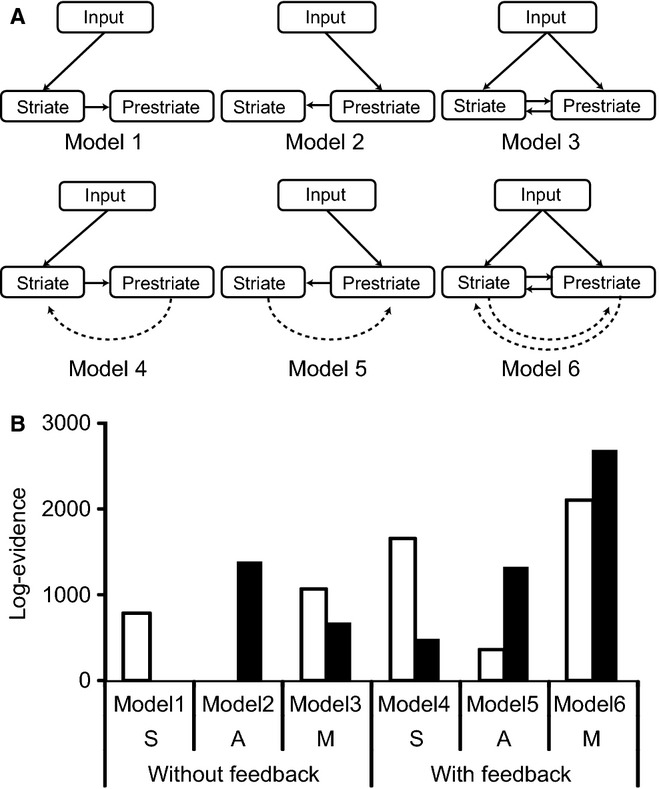
DCM analysis for the initial response. (A) Model specification for DCM analysis. Models were composed of two nodes (striate and prestriate cortex), feed-forward (solid arrows) and feedback (dashed arrows) connections. Models 1–3 had only feed-forward connections and models 4–6 included feedback connections as well. Models 1 and 4 represent sequential models, models 2 and 5 represent anti-sequential models, and models 3 and 6 represent multi-input models. (B) Bayesian model selection among DCMs for the six models. These values show the relative free energy approximation to the log-evidence compared to those for the worst model at group level. S, sequential model (input → striate → prestriate); A, anti-sequential model (input → prestriate → striate); M, multi-input model (input → striate and prestriate). White columns, lines; filled columns, rhomboids.

We constructed six different models (see Fig. 4A) using two nodes, striate and prestriate cortex. A sequential model (model 1) receives input directly into striate cortex followed by a forward connection to prestriate cortex. The anti-sequential model (model 2) is the reverse; input is received by prestriate cortex which has a forward connection to striate cortex. In the multi-input model (model 3) both striate and prestriate cortices receive input and each has a forward connection to the other. Each of these three primary models has a second version which includes feedback as well as forward connections (models 4, 5 and 6). These models were designed to distinguish between sequential and parallel processing strategies, enabling us to assess the probability of the competing hypotheses to account for how these brain areas interact.

We used the co-ordinates of the peak sources in striate and prestriate cortex, which we had already identified during our source analysis (Table 3). These co-ordinates were used for the equivalent dipole locations in the DCM analyses. The analysis time window was set between 0 and 52 ms after stimulus onset to isolate the initial responses and the input timing was set at 20 ms after stimulus onset. We used fixed-effects Bayesian model selection to compare our different hypotheses (models), pooling over the nasal and temporal quadrant presentations.

**TABLE 3:** MEG source peak co-ordinates (MNI) and significances

Response	*X*	*Y*	*Z*	*T*	*P* (peak)	
Quadrant						
Form						
Visual area					*P*_unc_	*P*_FWE_
Initial						
Nasal						
Lines						
Striate	4	−94	−2	5.01	3.91 × 10^−5^	
Prestriate	28	−86	−14	5.12	3.02 × 10^−5^	
Rhomboids						
Striate	2	−88	−6	4.22	2.33 × 10^−4^	
Prestriate	26	−88	−4	3.79	6.22 × 10^−4^	
Temporal						
Lines						
Striate	0	−86	−4	4.33	1.79 × 10^−4^	
Prestriate	−8	−88	−4	4.27	2.05 × 10^−4^	
Rhomboids						
Striate	4	−96	6	4.95	4.41 × 10^−5^	
Prestriate	−38	−34	50	3.74	6.89 × 10^−4^	
Main						
Nasal						
Lines						
Striate	2	−82	0	6.09		0.020
Prestriate	24	−92	−8	5.32		0.071
Rhomboids						
Striate	18	−96	2	5.50		0.076
Prestriate						
Temporal						
Lines						
Striate	0	−76	2	7.69		0.002
Prestriate	−10	−80	0	7.27		0.003
Rhomboids						
Striate						
Prestriate	−12	−90	−4	7.39		0.003

The visual zone was identified using the SPM Anatomy toolbox. *P*_unc._, probability uncorrected for multiple comparisons; *P*_FWE_, probability corrected family-wise for multiple comparisons. Uncorrected statistics are quoted here for the initial response to give locations for the suprathreshold activations reported in Fig. 2.

The six DCMs (each representing one of the models 1–6) were inverted (calculated) for each subject and for each of the four conditions (lines and rhomboids, nasal and temporal quadrants). The log-probability of the data (the likelihood) was then accumulated across all 20 subjects and combined between nasal and temporal quadrants to estimate model probability at the group level for each form (Garrido *et al*., 2007).

## Results

### Amplitude analysis

**Figure 5:**
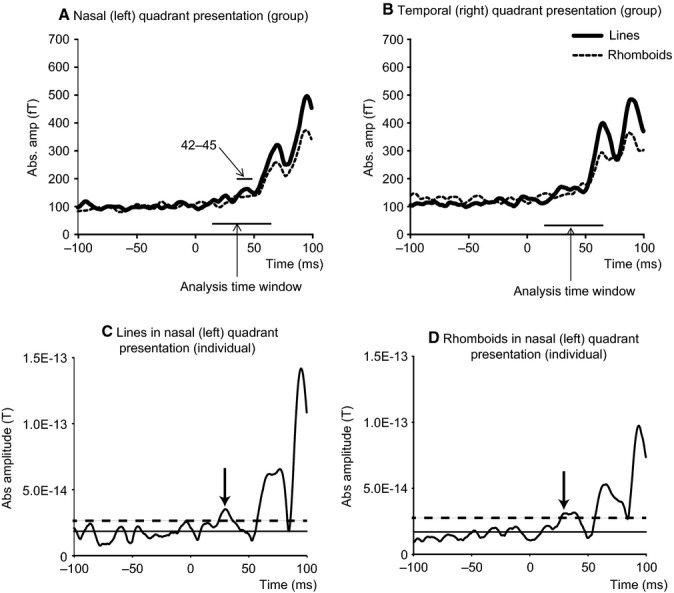
Absolute amplitude time-course around initial responses. (A and B) Differences in amplitude between lines and rhomboids averaged across 20 subjects. Solid and dashed lines show the absolute amplitude time-courses for lines and rhomboids respectively. (A) Absolute amplitude time-course of ERF for nasal (left) quadrant presentation measured by right occipital sensors. (B) Absolute amplitude time course for temporal (right) quadrant presentation measured by left occipital sensors. Numbers in figures indicate the time windows in which lines produced significantly stronger response than rhomboids. (C and D) Typical amplitude time-courses in individual subjects measured by whole occipital sensors. (C) Lines and (D) rhomboids in nasal (left) quadrant. The arrow indicates a peak of the initial response. Solid and dashed lines show the baseline average and average + 2 SD respectively.

The results, given in Fig. 5A, show that, for nasal stimulation, lines gave a stronger response than rhomboids at 42–45 ms, representing the initial response. In other words, a relatively simple form (lines) elicited a stronger response than a more complex form (rhomboids). For temporal quadrant stimulation, lines produced a stronger response but this did not reach significance (Fig. 5B). Although the peaks of initial responses produced by rhomboids in the two quadrants were not clear, their amplitudes were larger than their baseline averages + 2 SD, between 26 and 31 ms after stimulus onset, and we found initial peaks in individual subjects which met these criteria (Fig 5C and D, Table 1 and Data S1).

### Latency analysis

About half of the initial responses (across all subjects and conditions) were supra-threshold (amplitude > mean + 2 SD continued for four or more time bins) and the average latency was ∼40 ms (Table 1). A univariate two-way (quadrant × form) anova showed no significant main effect of either quadrant or form, but there was an interaction (Table 2). *Post hoc t*-tests showed that there was no significant difference in latency for either nasal or temporal quadrant presentations. Latencies for the main response are shown in Table 1.

**TABLE 2:** MEG latency analysis

	anova	*Post hoc t-*test	*P*
Initial response			
Main effect			
Form	*F*_1_ = 0.000		0.984
Quadrant	*F*_1_ = 2.900		0.097
Form × Quadrant	*F*_1_ = 4.950		0.032
Nasal quadrant		*t*_20_ = 1.825	0.083
Temporal quadrant		*t*_17_ = 1.375	0.187
Main response			
Main effect			
Form	*F*_1,19_ = 1.598		0.221
Quadrant	*F*_1,19_ = 0.213		0.650
Form × Quadrant	*F*_1,19_ = 6.846		0.017
Nasal quadrant			0.042
Temporal quadrant			0.379

Two way anova and *post hoc t*-tests for comparison of line and rhomboid and of nasal and temporal quadrant presentations.

### Source analysis

Estimated sources for lines and rhomboids were located in both striate and prestriate cortices, for both initial and main responses (Fig. 2, B3 and C3; Table 3) and displayed similar activation patterns for lines and rhomboids, and for nasal and temporal quadrant stimulation. Paired *t*-tests to compare the estimated sources for lines and rhomboids did not reveal any significant difference in initial or main activation produced by stimulation of the nasal and temporal quadrants by the two forms.

### DCM analysis

Bayesian model comparison, based on the increase in log-evidence across the six models, is shown in Fig. 4B. The multi-input model with feedback (model 6) is favoured for both lines and rhomboids. The anti-sequential model and the sequential model without feedback were the worst models of fit for line and rhomboid stimuli respectively. DCM is based on the concept of free energy which incorporates the available degrees of freedom in each model (Penny *et al*., 2004). In this way the complexity of each model is taken into account when selecting the best fit.

## Discussion

In this study we report four main findings related to the initial response component (∼40 ms) elicited by viewing forms. The response amplitude for lines is stronger than that for rhomboids (Fig. 5A), latencies for lines and rhomboids do not differ significantly (Table 1) and estimated sources for both lines and rhomboids are distributed in both striate and prestriate cortices (Table 3). DCM shows that a multi-input model is the best fit to explain our data, for both lines and rhomboids (model 6 in Fig. 4). In earlier evoked response studies, it was widely thought that the N75 response, with a latency ∼75 ms after stimulus onset, is the earliest visual evoked response component in the cortex (Nakamura *et al*., 1997; Tobimatsu & Celesia, 2006); however, some previous studies using MEG have shown that there is a very early response, ∼40–50 ms after stimulus onset, in V1 (P50 m, Nakamura *et al*., 1997; 37M, Inui *et al*., 2006) and V5 (ffytche *et al*., 1995). From the viewpoint of latency and source locations, the initial response we detected may in fact correspond to the 37M response which Inui *et al*. (2006) reported. Thus our findings shed light on the initial stage of form perception in the visual cortex.

These results speak in favour of a parallel strategy within one of the parallel processing systems of the visual brain, the form system. Our results gain strength from previous studies on the latency of activation for various visual areas, which have shown considerable overlap between different visual areas including between areas V1 and V2, thus casting doubt on strict hierarchical processing (Schmolesky *et al*., 1998; Schroeder *et al*., 1998; Nowak *et al*., 1995). However, these earlier results used flash stimuli, which may not always be the optimal stimuli for activating, with short latencies, areas that have concentrations of cells with particular and exigent requirements. An interesting example here is that of V5, which is heavily involved with visual motion (Zeki, 1974; Watson *et al*., 1993; Orban *et al*., 1995). The latency of activation in that area is 28–32 ms after onset of a fast moving stimulus (> 10 °/s) and 74 ms after onset of a slow moving stimulus (< 5 °/s; ffytche *et al*., 1995); hence V5 is activated before V1 with the former and after it with the latter, a finding that flashed stimuli would not have revealed. This has led to the suggestion that there is dynamic parallelism in activation of V5, depending upon the speed configuration of the stimulus (ffytche *et al*., 1995). Moroever, the work of Schoenfeld *et al*. (2003) shows that latency can be modulated by other factors such as attention. In the present study, we confined ourselves to stimuli composed of lines, which are known to activate OS cells in V1, V2 and V3 (Hubel & Wiesel, 1962, 1965; Zeki, 1978b; Yacoub *et al*., 2008; Aspell *et al*., 2010; Freeman *et al*., 2011; Tong *et al*., 2012), the three visual areas we were principally interested in for comparing directly the latency of activation produced by the same two stimuli. The results showed that simple forms (lines) produced a stronger earlier response than complex ones (rhomboids) with little difference in the latency of the initial response (40 ms), a finding that cannot be accounted for by feedback, which of course is known to play an important role in regulating the properties of cells in V1 (Lamme & Spekreijse, 2000; Murray *et al*., 2002). That this was not due to a failure of the MEG technique to detect differences is shown by its ability to detect a main response latency difference for nasal quadrant stimulation (see Table 2). Moreover, the DCM modeling suggests a strong preference for the parallel model in form perception, involving feed-forward as well as feedback connections between striate and prestriate cortex and with both areas receiving primary visual input.

Our results lead us to conclude that the perceptual hierarchy of forms is not mirrored by a sequential temporal hierarchy. This of course does not imply that a hierarchical strategy is not used within each area, as apparently is the case in V1 and V2 (see, for example, Alonso & Martinez, 1998; Martinez & Alonso, 2001) although even here a parallel operation may be at work, reflected in the fact that there is also little or no difference in onset and offset latencies for two categories of cell, the simple and complex ones, in the hierarchical chain (Bair *et al*., 2002). But our results here, as well as previous studies, suggest that if a hierarchical strategy is used, it must be used in parallel in each of the three areas, at least in the context of the stimuli that we have used.

### Possible confounds

There are three potential limitations to this study. (i) We only identified an initial response, at ∼40 ms, in about half of our measurements (Table 1). Although previous studies have shown that the early components of visual responses are not always identified, Shigeto *et al*. (1998) reported the N75 m response in 75% cases and Nakamura *et al*. (1997) also reported that they could only detect P50 m in a few case even with the use of powerful stimuli such as black-and-white checkerboard pattern reversals. This low detection ratio naturally raises suspicions about the response. However, we successfully estimated the sources in appropriate locations in a group level (between-subjects) analysis, which has a higher sensitivity than sensor-level analysis. Furthermore, we identified the laterality between hemispheres and amplitude differences between forms at the sensor level across subjects. In addition, the probability of observing an effect at *P *= 0.05 (i.e. greater than averages + 2 SD) in 10 out of 20 individuals is *P* = 1.3 × 10^−8^; in other words, the finding of an effect in only half of the subjects is very unlikely to have happened by chance. These findings indicate that there is a very early (initial) response even if peak detecting ratio in individual subjects was just over 50%. (ii) Inui *et al*. (2006) detected their very early response (37M) in all subjects in spite of using flash stimulation. They used a 37-channel axial-type first-order biomagnetometer but not of the whole head, and the intensity of their stimuli was 370 lux at eye position. Non-whole-head sensors allow for shorter distances between visual cortex and sensors while flash stimulation (which is very bright) activates a larger number of neurons in occipital cortex. These more favourable conditions might have allowed them to detect the very early response at sensor level at a higher rate than us. (iii) To resolve any doubt that the initial response might be a filter artifact produced by P100 m after using the 13-Hz high-pass filter, we also used a forward filter which does not produce such an artifact before P100 m. This filter erased the artifact just before P100 m (∼70 ms) but the initial response did not disappear. In addition, if the initial response was a filter artifact produced by the P100 m response, we would expect that a larger P100 m would produce an ‘initial response’ more often than a smaller P100 m. But there was no correlation (*P* = 0.47) between P100 m amplitude and ‘initial response’ occurrence in our data; mean amplitude of P100 m was 1.87×10^3^ fT when we detected the initial response and 2.03×10^3^ fT when we did not. Furthermore, contour maps for the initial response and the main response were different (Fig. 2). We conclude that the initial response is not a mere artifact of filtering related to P100 m.

Despite these potential limitations, our findings strongly suggest the existence of parallel processing streams in the visual form system.

## Abbreviations

DCM: dynamic causal modeling
ERF: event-related magnetic field
MEG: magnetoencephalography
OS: orientation-selective
SNR: signal-to-noise ratio

## Supporting Information

Data S1 Absolute amplitude time course of event related magnetic fields for rhomboid stimuli in individual subjects.
