# Supplementary material for: Parallelism in the brain's visual form system
Source: Eur J Neurosci. 2013 Oct 7;38(12):3712–20. doi: 10.1111/ejn.12371 (PMC3995019; doi:10.1111/ejn.12371)
Supplement: Data S1 — Absolute amplitude time course of event related magnetic fields for rhomboid stimuli in individual subjects. [file ejn0038-3712-sd1.docx]

**Supplementary document**

**Absolute amplitude time course of event related magnetic fields for rhomboid stimuli in individual subjects**


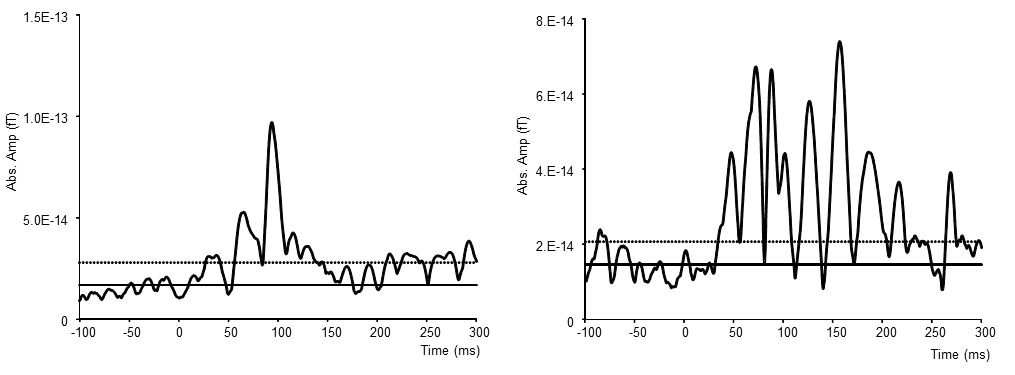


Subject01 Rhomboids in nasal quadrant Subject02 Rhomboids in temporal quadrant


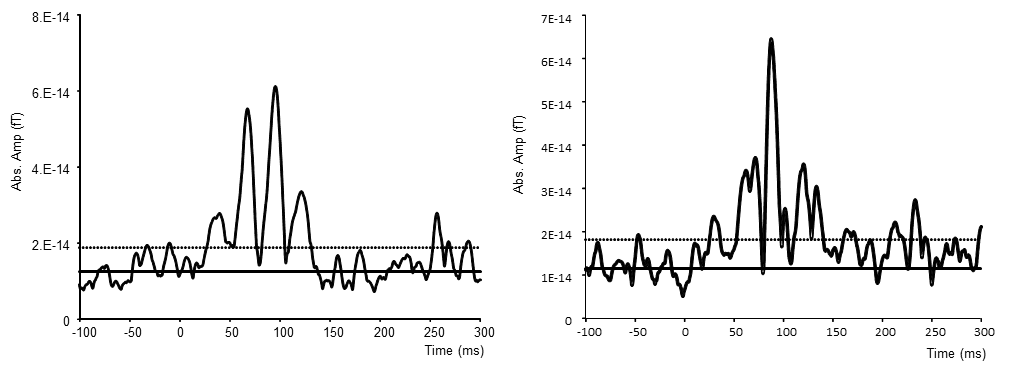


Subject03 Rhomboids in nasal quadrant Subject03 Rhomboids in temporal quadrant


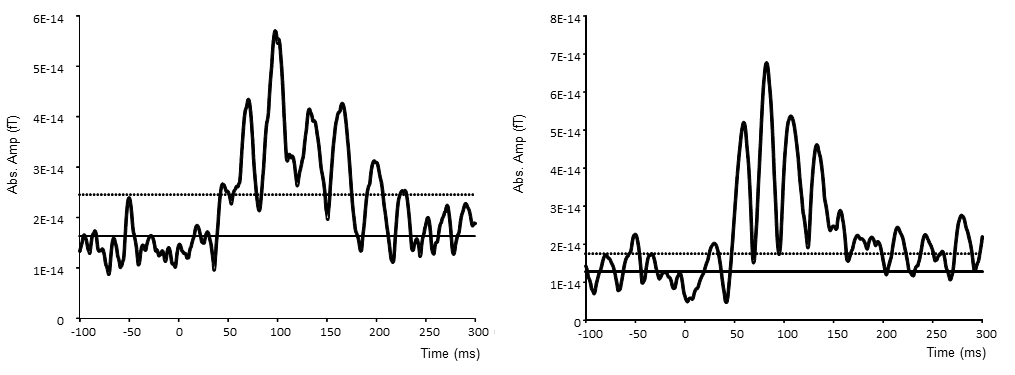


Subject04 Rhomboids in nasal quadrant Subject05 Rhomboids in temporal quadrant


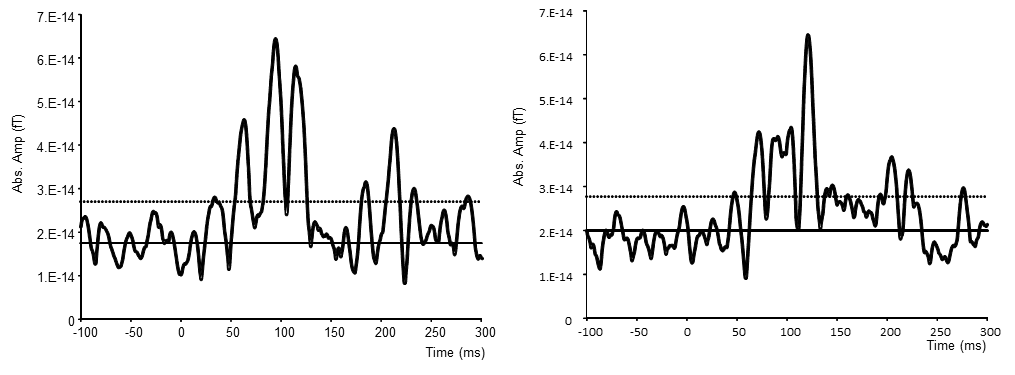


Subject06 Rhomboids in nasal quadrant Subject06 Rhomboids in temporal quadrant


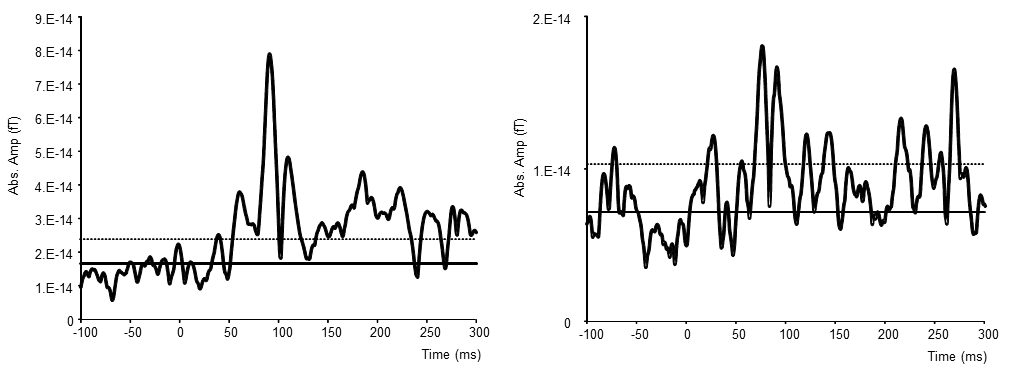


Subject07 Rhomboids in nasal quadrant Subject08 Rhomboids in nasal quadrant


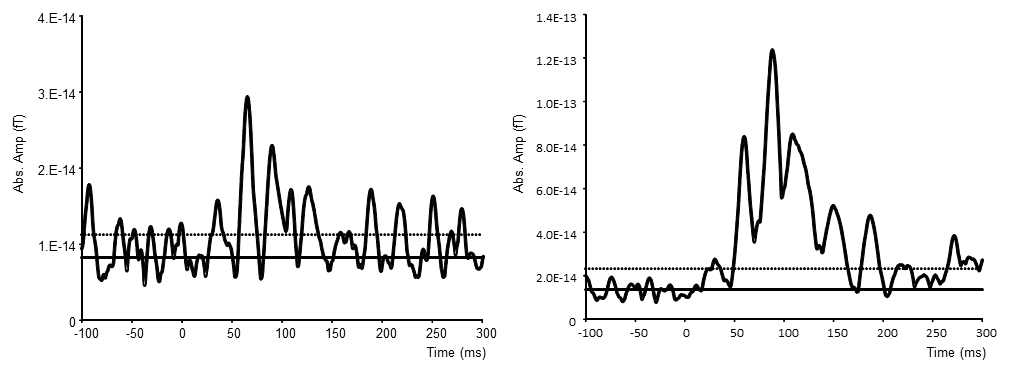


Subject08 Rhomboids in temporal quadrant Subject09 Rhomboids in temporal quadrant


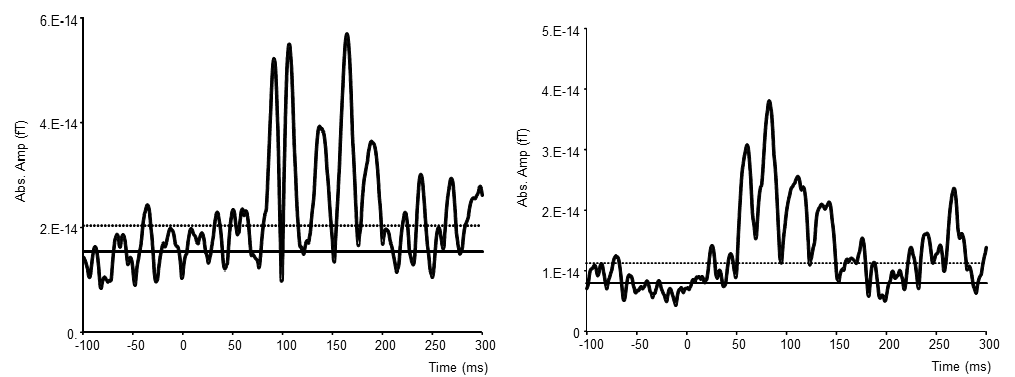


Subject11 Rhomboids in nasal quadrant Subject13 Rhomboids in temporal quadrant


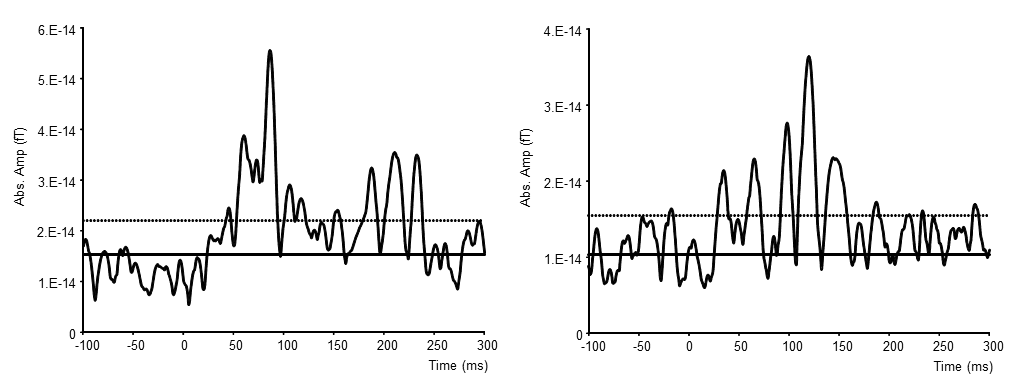


Subject14 Rhomboids in temporal quadrant Subject15 Rhomboids in temporal quadrant


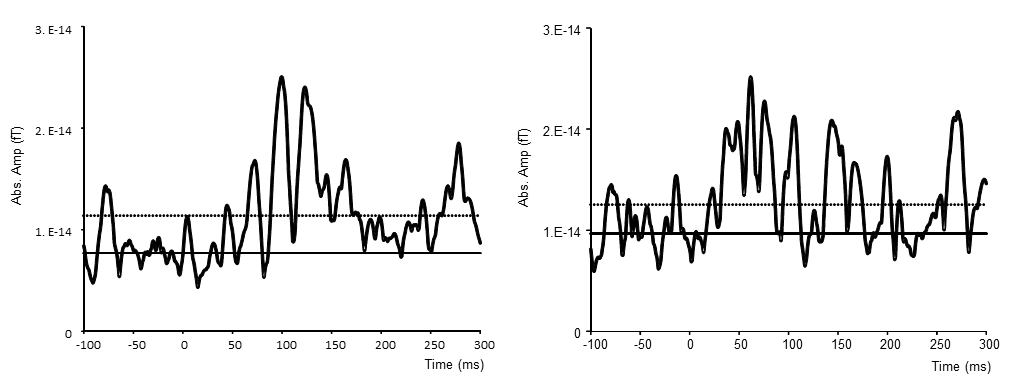


Subject18 Rhomboids in nasal quadrant Subject20 Rhomboids in temporal quadrant
